# Contrasting diversity patterns between microeukaryotic and prokaryotic communities in cold-seep sediments

**DOI:** 10.1093/ismeco/ycaf002

**Published:** 2025-01-08

**Authors:** Zhimeng Xu, Jiawei Chen, Wenzhao Liang, Zhao Liang Chen, Wenxue Wu, Xiaomin Xia, Bingzhang Chen, Ding He, Hongbin Liu

**Affiliations:** Department of Ocean Science, Hong Kong University of Science and Technology, Hong Kong, 000000, China; Department of Ocean Science, Hong Kong University of Science and Technology, Hong Kong, 000000, China; Department of Ocean Science, Hong Kong University of Science and Technology, Hong Kong, 000000, China; Department of Ocean Science, Hong Kong University of Science and Technology, Hong Kong, 000000, China; State Key Laboratory of Marine Resource Utilization in South China Sea, Hainan University, Haikou, 570000, China; Carbon Neutral Innovation Research Center and State Key Laboratory of Marine Environmental Science, Xiamen University, Xiamen, 361102, China; Key Laboratory of Tropical Marine Bio-resources and Ecology, South China Sea Institute of Oceanology, Chinese Academy of Sciences, Guangzhou, 510000, China; Department of Mathematics and Statistics, University of Strathclyde, Glasgow, G1 1XH, United Kingdom; Department of Ocean Science, Hong Kong University of Science and Technology, Hong Kong, 000000, China; Department of Ocean Science, Hong Kong University of Science and Technology, Hong Kong, 000000, China

**Keywords:** cold seep, metabarcoding, microbial diversity, ecological process, relative activity

## Abstract

Cold seeps are hotspots of biodiversity. However, the quantification of the microbial diversity, particularly that of microeukaryotes, remains scarce and little is known about the active groups. In this study we investigated the diversity and activity of prokaryotes and microeukaryotes in the Haima cold seep sediments in the northern South China Sea using both DNA (whole community) and RNA (active community) signatures. We found that, in general, prokaryotes had lower diversity in the seep sediment than in non-seep regions while microeukaryotes showed the opposite pattern. This finding could be explained by the dominance of homogeneous selection in the prokaryotic community while microeukaryotic communities were less affected by environmental selection, harboring high richness of abundant groups in the seep regions. The compositional difference between DNA and RNA communities was much larger in microeukaryotes than prokaryotes, which could be reflected by the large number of inactive microeukaryotic taxa. Compared to the whole community, the seep-active groups, e.g. among microeukaryotes, *Breviatea, Labyrinthulomycetes*, and *Apicomplexa* were more sensitive to and directly influenced by environmental factors, suggesting their pivotal roles in ecosystem biodiversity and functions. This study provides insight into the distinct diversity patterns and regulating mechanisms that occur between prokaryotic and microeukaryotic communities in cold-seep sediments, deepening our understanding of microbial ecology in deep-sea extreme habitats.

## Introduction

Cold seeps are unique seafloor ecosystems fuelled by chemical energy originating from microbial transformation of methane, sulphide, and other hydrocarbons. Widely distributed in the global ocean, cold seeps act as major methane (and carbon) sinks through methane oxidation by microorganisms in sediments, thereby preventing deep-layer methane from entering the atmosphere [1]. Due to their patchy distribution and spatiotemporal dynamics of energy (e.g. methane) supplied from deep layers, cold seeps are highly fragmented and isolated from surrounding seafloor environments, acting as island-like habitats that offer unique niches for organisms [2–4]. Despite the extreme environmental conditions, such as high sulphide concentrations, oxygen depletion (occurring only in a few millimeters to centimeters of the sediment surface), and high pressure, cold seeps harbor thriving and diverse life, indicating their importance in the evolution, diversification, and dispersal of species and the connectivity of ecosystems [3, 5]. Microorganisms in cold seeps, including both prokaryotic and eukaryotic communities, contribute significantly to the global biogeochemical cycles. For instance, *Bacteria* and *Archaea* play key roles in the main functions of cold seeps, e.g. hydrocarbon degradation, sulphide production and consumption, and chemosynthetic CO_2_ fixation, as well as controlling the flux of methane from the sediments into the water column [3]. Specialized microbial eukaryotic assemblages with possible parasitic or symbiotic trophic status may exist in the microbial niches of cold seep sediments, serving as primary producers and consumers and providing important links to higher trophic levels [2, 6].

Thanks to modern sequencing technology, studies over the past two decades have shown that cold seeps are hotspots not only for large animals (metazoans) but also for microbes, including viruses [2, 7]. Typically, the prokaryotic communities in cold seeps are dominated by aggregates of syntrophic partners of anaerobic methanotrophic archaea (ANME) and sulphate-reducing bacteria (SRB) [1, 8–10]. Along with other abundant groups such as *JS1*- and Chloroflexi-related bacteria, these organisms are responsible for the majority of the production and consumption of methane, sulphate, and hydrocarbons, thus regulating the biogeochemical cycles in cold seeps [11, 12]. A global study has shown that methane seep communities had moderate levels of prokaryotic richness compared to other seafloor ecosystems (with the highest richness at the deep-sea surface and the lowest richness in hydrothermal vents), with highly local diversification of ANME [3]. SRBs were found to have vast biodiversity with different metabolic rates and lifestyles in the cold seeps [13–15]. A study across nine different cold seeps in the Eastern Mediterranean showed that bacterial communities differed considerably on spatial scales of only tens to hundreds of meters, suggesting that cold seeps contribute substantially to the microbial diversity of the deep sea [16]. However, there is still a lack of quantitative assessments of prokaryotic diversity at different scales in cold seeps, which are essential for the understanding of the roles of this unique habitat in shaping biodiversity.

Microeukaryotes (or microbial eukaryotes, mainly including protists and fungi), representing the eukaryotic part of microbes, are diverse and abundant in most ecosystems [17, 18]. With different trophic modes, such as autotrophy (e.g. green algae), heterotrophy (e.g. ciliates), osmotrophy (e.g. fungi), parasitism (e.g. apicomplexans), and mixotrophy (e.g. dinoflagellates), microeukaryotes play crucial roles in food webs and biogeochemical cycles in marine ecosystems [18–20]. However, compared to prokaryotes and large fauna (animals), microeukaryotes are much less studied in cold seeps, with sporadic reports of a few dominant groups such as ciliates and fungi and their novel lineages [21–24]. Our recent study revealed that microeukaryotic diversity, from both local and regional scales, was higher in cold-seep sediments than sediments in non-seep regions, with representative groups such as *Apicomplexa* [25]. A previous study showed that the diversity and community structure of microeukaryotes were affected by substrate type, seep activity, and sulphide concentration in the methane seep ecosystem off the coast of Oregon (United States) [2].

Disentangling the ecological process underlying diversity and community structure is a key issue in microbial ecology [26]. “Marine protists are not just big bacteria,” [27] and their complicated behaviour and ecological traits may greatly influence the community assembly processes [27–29]. This characterization is supported by findings that microeukaryotes and bacteria under the same environment can be regulated by different dominant processes. For instance, in a study by Wu *et al*. (2018) [30] conducted at the East China Sea, protistan communities were more dominated by environmental selection than dispersal limitation, whereas bacteria displayed the opposite pattern. In a global study of the pelagic ocean by Logares *et al*. (2020) [31], picoeukaryotic communities were predominated by dispersal limitation while bacterial communities were regulated by the combined effects of dispersal limitation, selection, and drift. This discrepancy can be attributed to the fact that relative contributions of ecological processes are largely affected by habitat type (e.g. environmental factors) and sampling scales (e.g. spatial) [32, 33], which have been much less studied in marine sediments than in water columns. Given the unique environmental conditions in cold-seep sediments, we hypothesized that the diversity patterns of microeukaryotic and prokaryotic communities may have been shaped by distinct ecological processes that have not yet been adequately tested.

Another issue regarding the use of environmental DNA in studying microbes is the uncertainty of the metabolic activity of organisms, because extracellular DNA and DNA derived from dead or dormant cells can persist over a long period, potentially leading to inaccurate estimation of diversity and its relationships to environmental factors. This issue may be more severe in marine sediments, which are depositing places for dead organisms from upper layers, especially for deep-sea cold-seep regions with high sedimentation rates [34] and low temperatures that extend DNA preservation. On the other hand, amplification of RNA (complementary DNA [cDNA]) is performed on active cells, which can detect the active taxa in the community and be used to identify the taxa that survive in different environmental conditions. However, several critical limitations in using RNA to investigate microbial communities require careful consideration [35, 36]. For instance, ribosomal RNA (rRNA) is the major component of total RNA, but both active and dormant cells may have high numbers of ribosomes (where rRNA comes from), making the revealed community putatively active; rRNA concentration is not constantly related to growth rate, obscuring its correlation with the abundance or relative abundance of microbial taxa [35, 36]. In addition, since RNA are very easily degraded even in hours, loss of RNA may happen randomly and inevitably, which may have great impacts on the evaluation of microbial biodiversity, especially for rare species. Studies have shown that combining both DNA and RNA signatures can provide comprehensive results in revealing the whole community structure and putatively active groups, along with the relative activity (ratio of relative abundance of RNA in relation to DNA) of each taxon [37], characteristics that are crucial to understanding the ecological processes [30] and environmental factors regulating microbial communities [38]. However, to date the combination of DNA and RNA signatures has been used only infrequently for studying the diversity and community structures of microorganisms in cold seeps, and less is known about the active microorganisms.

In this study, we investigated the microbial communities, including both microeukaryotic and prokaryotic communities, in the sediments of the Haima cold seep, a typical active methane seep region in the South China Sea [39]. By employing both DNA- and RNA-based metabarcoding, we quantified microbial diversity at multiple scales (α, β, and γ), and compared these findings with the diversity of non-seep regions. We described the structure of the whole community (DNA) and putatively active components (RNA) and identified the seep-active groups based on their relative activities. In particular, to offer a comprehensive understanding of the relationships between microbes and environmental factors in cold seeps we measured a large number of environmental parameters that have been inadequately examined in most previous studies, including the components and characteristics of organic matter closely related to microeukaryotic diversity and community composition [40, 41]. Our main aims were to study the diversity patterns and regulating mechanisms of microbial communities in the cold-seep sediments, with identification of the putatively active groups. We hypothesized (1) that microeukaryotes and prokaryotes had different diversity patterns, which were regulated by different ecological processes in cold seeps; and (2) that active microbial groups contributed significantly, even more than the whole community, to the biogeochemical cycles in cold seeps.

## Materials and methods

### Sampling collection and measurement of environmental factors

**Figure 1:**
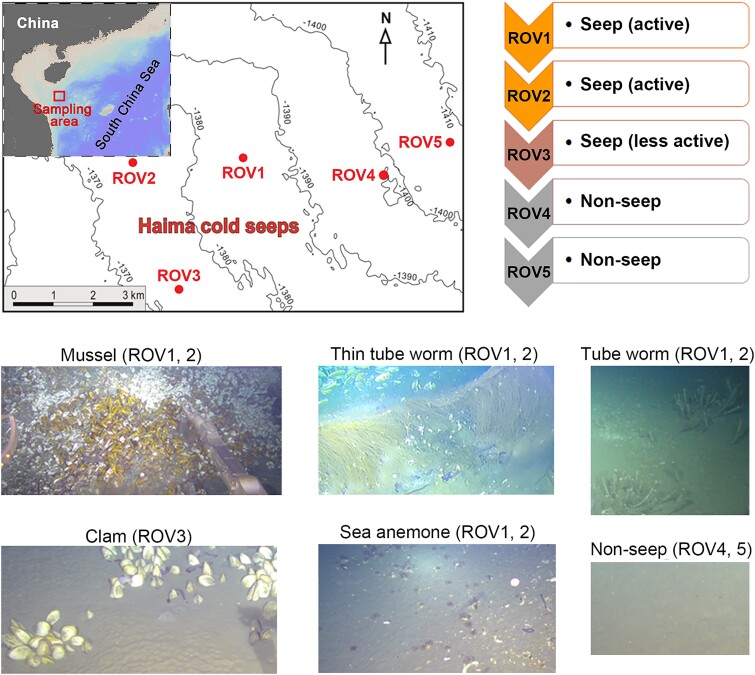
Sampling maps and representative habitats.

Sediment samples were collected from the Haima cold seep (16.72°N, 110.46°E, bottom depth of ~1400 m) at the northern South China Sea during cruise HYDZ6–202102 on R/V “Haiyangdizhi VI” in May 2021 (Fig. 1). Sediment push cores were retrieved using the remotely operated underwater vehicle (ROV) from both seep and non-seep regions with different biological and chemical activities: (1) active seep regions, represented by ROV1 (9 sediment cores were collected) and ROV2 (9 sediment cores), mainly mussel beds (massive mussels and arthropods, e.g. crab and shrimp, and visible bubbling of seepage showing high activity) and a few other habitats dominated by thin tube worms or sea anemones; (2) less active seep regions, represented by ROV3 (10 sediment cores), dominated by clam beds with patchy distribution of *Archivesica* spp. (*Bivalvia*: *Vesicomyidae*) [39]; (3) non-seep regions, represented by ROV4 (3 sediment cores) and ROV5 (2 sediment cores), which were nearby marine sediments (several kilometers from ROVs 1–3) with a flat seafloor and few megafauna.

Sediment cores, with depths ranging from 0 to 70 cmbs (centimeters below surface, unevenly distributed among sediment cores) were immediately stored at −80°C (or −20°C) on board. In the laboratory, subsampling was conducted at every 5 cm for each sediment core and used for further procedures. We admit that the sampling process (e.g. from sediment to surface water) conducted on board may induce significant changes of messenger RNA (mRNA) expression (e.g. due to changes of temperature, oxygen, and pressure); however, this process had minor effects on the ribosomal RNA (rRNA) (used for the RNA communities in our study), as rRNA is more stable than mRNA and usually predominates in the total RNA [35].

We measured a total of 19 environmental parameters, including inorganic and organic matter and stable isotopes, to characterize the habitat conditions and for further correlation analyses. In detail, pore water for each sample was extracted from the sediment cores using Rhizon moisture samplers and measured for the following chemical factors. Methane (CH_4_) concentration was measured with gas chromatography (Agilent 6850, Agilent Technologies, United States); sulphate (SO_4_^2−^) and ammonium (NH_4_^+^) were measured with an ion chromatography system (ISC-1000, Thermo Scientific, United States). Sulphide (S^2−^) and phosphate (PO_4_^3−^) were measured using a discrete autoanalyzer (Smartchem200, Alliance, France). Concentrations of dissolved inorganic carbon (DIC) were tested by a mass spectrometer Delta V Advantage (Thermo scientific, United States) with PoraPlotQ column (Agilent Technologies, United States).

Water-extractable organic matter was used as a proxy of dissolved organic matter (DOM) in the sediment samples. Briefly, 0.4 mg of freeze-dried, homogenized sediment was added to 40 ml of MiliQ H_2_O in a polypropylene tube and shaken vigorously for 24 hours in the dark, before being centrifuged for 30 minutes at 3600 rpm. Supernatants were filtered through 0.45-μm cellulose acetate syringe filters (Microanalytix Pty Ltd, Australia). Then, the UV-visible absorbance and three-dimensional excitation emission matrix of the extracts were measured using an Aqualog absorption fluorescence spectrometer (Horiba, Japan) at 3-nm intervals with a 1-cm quartz cell. Excitation wavelengths ranged from 250 to 500 nm and emission wavelengths ranged from 250 to 600 nm. Fluorescence signals were corrected for Raman scattering and inner filter effects [42]. We measured the compositions (e.g. humic-like compounds “DOM_a,b,c,m” and less-degraded peptide material “DOM_t”) and characters (e.g. biological index [BIX], humification index, spectral slope ratio, and fluorescence index) of water-extractable organic matter, with detailed descriptions of these parameters shown in Table S1.

Stable isotope analyses were performed with sediments treated with an excess volume of hydrochloric acid (6 M) to remove carbonates and then dried (at 55°C for 48 hours). The isotopic ratios of dried samples were determined by use of an elemental analyzer (EA-EuroVector) connected to a stable isotope ratio mass spectrometry system (Nu Perspective) and reported in standard delta (δ) notation (δ^13^C or δ^15^N), defined as parts per thousand (‰) deviation from a standard: δ^13^C or δ^15^N = ((R_sample_: R_standard_) – 1) × 1000) [43]. Percentages of organic carbon (%OC) and total nitrogen ( %TN) were also calculated for each sample.

### Nucleic acid extraction, cDNA synthesis, polymerase chain reaction, and sequencing

DNA and RNA were extracted from sediment samples with a DNeasy PowerSoil Pro Kit (QIAGEN, United States) and an RNeasy PowerSoil Total RNA Kit (QIAGEN, United States), respectively, following their user’s protocols. Quality and concentration of extracted DNA were measured by use of a Qubit dsDNA Assay Kit in a Qubit 2.0 Fluorometer (Life Technologies, United States). The RNA extracts (partial, according to their concentrations) were converted into cDNA using a Vazyme HiScript II 1st Strand cDNA Synthesis Kit (Vazyme Biotech Co., Ltd, China) following the manufacturer’s protocol.

To reveal the microeukaryotic communities, polymerase chain reaction (PCR) was conducted with barcoded universal primers targeting the hypervariable V4 region of the 18S rRNA gene: 528F (5′-GCGGTAATTCCAGCTCCAA) and 706R (5′-AATCCRAGAATTTCACCTCT) [44] for both DNA (extracted) and cDNA (synthesized from RNA). For prokaryotic communities, PCR reactions were performed with the 515F/806R primer pair (forward: GTGCCAGCMGCCGCGGTAA; reverse: GGACTACHVGGGTWTCTAAT) [45], which was efficient in the detection of both bacteria and archaea. Both DNA and cDNA samples were amplified separately, with the reaction conditions following those of the microeukaryotes mentioned above. PCR was prepared with a mixture of 2.5 μl of 10× PCR buffer, 0.75 μl of 10 mM MgCl_2_, 0.5 μl of 10 mM dNTP mix, 0.5 μl of each primer (10 μM), and 1 U of Invitrogen Platinum Taq DNA polymerase (Life Technologies, United States). The PCR reaction conditions were set as an initial denaturation of 3 minutes at 94°C, followed by 30 cycles of 30 seconds at 94°C, 30 seconds at 60°C, 1 minute at 72°C, and a final cycle of 5 minutes at 72°C. All PCR reactions were prepared in triplicates, pooled together into a library, and sequenced by a Hiseq 2500 System (Illumina, United States) with 2 × 250–bp paired-end read configurations.

### Raw sequencing data processing

Raw sequencing reads were processed using the online pipelines of QIIME2 (version 2023.5.1) (https://docs.qiime2.org/) [46]. In brief, after removal of the barcode and primer, paired reads were imported into QIIME2, checked for sequence quality after demultiplexing, and processed with DADA2 to remove contamination, trim reads, correct errors, merge read pairs, and remove chimeras (i.e. mismatched reads). Representative amplicon sequence variant (ASV) sequences and their abundances were extracted from a feature table. Phylogenetic trees, both rooted and unrooted, were built with representative ASVs based on their nucleic acid sequences and used for further analysis when their phylogenetic distances were needed. A naive Bayes classifier was trained with 16S rRNA and 18S rRNA sequences extracted from SILVA v138.2 database [47] and Protist Ribosomal Reference (PR^2^) database [48], respectively, according to the PCR primers used here. Then, the representative ASV sequences were classified with detailed taxonomy information using the trained classifier. ASVs with taxonomic assignment of metazoans and land plants were removed from the microeukaryotic communities while ASVs belonging to mitochondria and chloroplasts were removed from the prokaryotic communities.

### Community construction and diversity estimation

The following analyses were conducted with different packages in R software (version 4.1.1) [49]. Microbial community structures, from both DNA and RNA signatures, were characterized by the relative abundance of sequences at the class or phylum (for microeukaryotes) levels, highlighting the dominant groups. Abundant groups, at the class or phylum level, were identified with mean relative abundances >0.1% across the regional community [50]. Differences in the distributions of abundant microbial groups between seep and non-seep samples were determined with the STAMP (statistical analysis of metagenomic profiles) graphical software package, with the significance corrected by “Bonferroni” method [51]. At the ASV level, community similarity between two samples was calculated using the Bray–Curtis distance (“vegdist” function in “vegan” package) [52] and visualized on a nonmetric multi-dimensional scaling map using the first three dimensions (“monoMDS” function in “vegan” package) in a 3D format (“scatter3d” function in “car” package) [53]. The effects of habitat type (i.e. ROVs 1–5) and sediment depth (i.e. 0–70 cmbs) on community structure were tested with analysis of similarity (ANOSIM) (“anosim” function in “vegan” package).

To ensure equal comparisons of diversity, we kept only the DNA samples that had a corresponding RNA community counterpart, with depth ranging from 0 to 10 cmbs. We focused on the comparison of diversity between habitats (i.e. seep versus non-seep) because effects from sediment depth on the communities were minor (from the results of above ANOSIM analysis).

Microbial diversity was calculated and compared at α, β, and γ scales. Here, α-diversity was defined by ASV richness (and other indices) of a local community in each layer of a sediment core and β-diversity was compared between two local communities within or between sediment cores, while γ-diversity was estimated as the total richness of ASVs within a habitat (ROV). Indices of richness (i.e. observed ASV number in a local sample), Chao1 (an estimation of diversity with the consideration of singletons and doubletons), Faith’ PD (phylogenetic diversity), and Pielou’s evenness index were calculated for α-diversity, while Bray–Curtis distances were used for comparison of β-diversity [54–56]. To avoid pure sampling effects on comparing γ-diversity [57, 58], especially in our study with the unequal numbers of samples among ROVs, we plotted an ASV accumulation curve for each habitat (“specaccum” function in package “vegan,” method = “random”) to compare the γ-diversity with an equal number of samples among habitats. The Wilcoxon test (“wilcox.test” function in R) was conducted to determine the significant difference of each diversity index between seep (ROV1–3) and non-seep regions (ROV4–5).

### Environmental factors and ecological processes regulating microbial diversity

Effects of environmental factors on microbial richness were analyzed by Spearman correlation and significant correlations were shown using by “ggplot2” package in R [59]. Redundancy analysis (RDA) was used to study the influence of environmental factors on the compositional (ASV level) variations of microbial communities (“rda” function in “vegan” package). Total explanation from environmental factors and the relative contribution of each factor on community variations were quantified by the “env.fit” function.

Null models were used to estimate the relative contribution of ecological processes on regulating the microbial community β-diversity [60]. Before making these estimates, we tested the phylogenetic signal to determine whether we could use phylogenetic turnover to make ecological inferences in our metacommunity system [60, 61]. We found significantly positive correlations at short phylogenetic distances, showing that phylogenetic signals existed for all types of communities (except microeukaryotic RNA communities) (Fig. S1). Then, the phylogenetic turnover using the abundance-weighted β-mean nearest taxon distance (βMNTD) metric was measured, which quantifies the mean phylogenetic distances between two evolutionary-closest ASVs in two communities:


$$ {\beta} \mathrm{MNTD}=0.5\left[\sum_{{\mathrm{i}}_{\mathrm{k}}=1}^{{\mathrm{n}}_{\mathrm{k}}}{\mathrm{f}}_{{\mathrm{i}}_{\mathrm{k}}}\min \left({\Delta }_{{\mathrm{i}}_{\mathrm{k}}{j}_{\mathrm{m}}}\right)+\sum_{{\mathrm{i}}_{\mathrm{m}}=1}^{{\mathrm{n}}_{\mathrm{m}}}{\mathrm{f}}_{{\mathrm{i}}_{\mathrm{m}}}\min \left({\Delta }_{{\mathrm{i}}_{\mathrm{m}}{j}_{\mathrm{k}}}\right)\right], $$

where ${f}_{i_k}$ is the relative abundance of ASV *i* in community *k*, ${n}_k$ is the number of ASVs in *k* and min(${\Delta }_{i_k{j}_m}$) is the minimum phylogenetic distance between ASV *i* from community *k* and all ASVs *j* from community *m*. The null model expectation was performed using 999 randomizations, and deviation between the observed βMNTD and the mean null model distribution is calculated as β-nearest taxon index (βNTI). A significant deviation (i.e. |βNTI| > 2) indicates the dominance of selection processes: βNTI < −2 indicates significantly less phylogenetic turnover than expected (i.e. homogeneous selection), whereas βNTI > 2 indicates significantly more phylogenetic turnover than expected (i.e. heterogeneous selection). Low deviation (i.e. |βNTI| < 2) indicates that the β-diversity of communities could be structured by stochastic processes such as dispersal and ecological drift. βNTI was calculated between any two communities in an ROV and compared between ROVs.

Niche breadth is a key factor influencing the relative importance of ecological processes in microbial communities. An organism with a wider niche breadth can be expected to be more metabolically flexible at the community level [62]. In this study we estimated the niche breadth of taxa within each ROV, using the Levins’ niche breadth (*B*):


$$ {B}_j=1\Big/ \sum_{i=1}^N{P}_{ij}^2, $$

where *B_j_* represents the habitat niche breadth of operational taxonomic unit (OTU) j in a metacommunity; *N* represents the total number of local communities in the metacommunity; and *P_ij_* is the proportion of OTU *j* in local community *i*. The calculation of niche breadth was conducted using the “niche.width” function in the “spaa” package [63].

### RA and seep-active groups

The RA of each ASV was calculated as the RNA:DNA ratio based on its relative abundance (in the rarefied tables) in the RNA and DNA communities. This ratio was used as a proxy of relative metabolic activity [64]. While “phantom taxa” (only detected in RNA communities) were removed from the calculation of RA, we kept the ASVs only detected in DNA communities (i.e. RA = 0) which could indicate the status of microorganisms (dead or alive) and their contributions to biogeochemical cycles in the cold-seep sediments, even if they are dead.

RA was calculated for the ASVs within the abundant groups of microbial communities. Seep-active groups were identified by conducting a Wilcoxon test of RA between seep and non-seep samples. Microbial groups with significantly higher RA in the seep than non-seep regions were identified as seep-active groups and used for further analysis. Effects of environmental factors on the richness and relative abundance of seep-active groups were analyzed by Spearman correlations and Mantel tests, respectively, and compared to their effects on the whole community.

### Structural equation modeling

To understand how environmental factors regulate microbial diversity in the cold seep, we employed structural equation modeling to quantify their relative contributions. Here, the structural equation models (SEMs) included three groups of factors: environmental factors, seep-active groups, and the whole community and were built for both microeukaryotes and prokaryotes, with their DNA and RNA communities, respectively (i.e. generating four SEMs), to compare the different regulating mechanisms between them.

SEMs were built with the “lavaan” package in R [65]. Only data from seep samples were used. We tested the normality and homogeneity of variance of the data using the Shapiro–Wilk and Levene’s tests, respectively. If the raw data were not normally distributed, we applied log-transformation. The model was accepted when the *P*-value associated with the chi-square value is greater than .05. Indices such as the goodness of fit index (GFI), comparative fit index (CFI), and root mean square error of approximation (RMSEA) were used to evaluate the model fit. The model is considered a good fit when those indices meet these criteria: GFI ≥ 0.9, CFI ≥ 0.95 and RMSEA ≤ 0.05).

## Results

### Environmental characteristics

In general, the seep region had higher concentrations of methane (CH_4_), sulphide (S^2−^), ammonium (NH_4_^+^), DIC, percentage of organic carbon (%OC), and percentage of total nitrogen (%TN), as well as a greater composition of dissolved organic matters (DOM_a, b, c, m, and t) and BIX of DOM (DOM_BIX) compared to the non-seep region (Wilcoxon test, *P* < .001) (Fig. S2, Metadata file 1). In particular, the highest concentrations of CH_4_ (4993.39 ± 2545.32 mg/kg) and DIC (17.89 ± 5.95 mmol/l) were detected in ROV1. The opposite pattern was detected in the concentration of SO_4_^2−^, where the ROV1 had the lowest value of 1040.12 ± 691.24 mg/l. The active seeps (ROV1 and 2) had much lower values of δ^13^C (−28.46 ± 4.97‰, Vienna PeeDee Belemnite [VPDB]) and δ^15^N (2.62 ± 1.21‰, VPDB) compared to non-seep regions (δ^13^C: −20.89 ± 0.36‰, δ^15^N: 4.94 ± 0.33‰, VPDB), indicating their biogenic origins.

### Microbial community structure

In total, 283 microeukaryotic communities (consisting of 29 480 ASVs and 11 402 388 sequences) were constructed by sequencing of 18S rDNA V4 region, including 210 DNA communities and 73 RNA communities, after removal of metazoan and terrestrial plant sequences. For prokaryotes, 303 communities (consisting of 107 915 ASVs and 23 316 406 sequences) were revealed, including 210 DNA communities and 93 RNA communities, after removal of chloroplast and mitochondria sequences.

**Figure 2:**
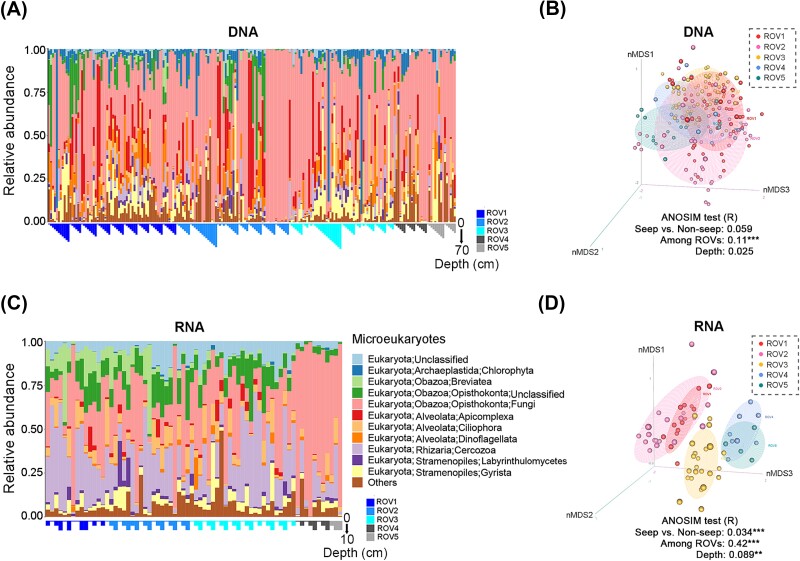
Community structure of microeukaryotes. Relative abundances of sequences were shown at the group level (class or phylum) for both DNA and RNA communities, with a shared legend of microeukaryotic community compositions (A and C). Community compositional difference (amplicon sequence variant [ASV] level) among remotely operated vehicle habitats (ROVs) were shown in the non-metric multidimensional scaling (NMDS) maps and tested for the effects from habitat type (i.e. seep and non-seep), sub-habitat (i.e. ROVs) and sample depth by analysis of similarity (ANOSIM). Each dot represents a community. A higher ANOSIM-R value indicates a larger effect from the factor on the community differences (B and D).

Microbial community compositions, including both microeukaryotes and prokaryotes, were compared between DNA and RNA signatures and between seep and non-seep regions. For microeukaryotes (18S), a substantial difference of community structure was observed between the DNA and RNA communities (ANOSIM-R = 0.41, *P* < .001), indicating the inconsistency of the whole community and the putatively active community (Fig. 2). Sequences from *Chlorophyta* (Archaeplastida) had remarkable proportions in the DNA communities (2.64%, on average) but were nearly undetected in the RNA communities, indicating their dead or inactive status. In contrast, much higher percentages of sequences from *Breviatea* (Obazoa), unclassified *Opisthokonta* (Obazoa), and *Cercozoa* (Rhizaria) were detected in the RNA communities (3.11%, 4.87%, and 13.31%, respectively) than the DNA communities (0.34%, 2.62%, and 1.76%, respectively), indicating their active status. Habitat type, especially ROV difference, had much higher impacts on the microeukaryotic RNA communities (ANOSIM-R = 0.42, *P* < .001) than the DNA counterpart (ANOSIM-R = 0.11, *P* < .001). Besides, a few microeukaryotic groups, such as *Apicomplexa* (Alveolata) and *Breviatea* (Obazoa), had higher relative abundances in the seep region than the non-seep region in both DNA and RNA communities (*P* < .001, by Wilcoxon test) (Fig. S3).

**Figure 3:**
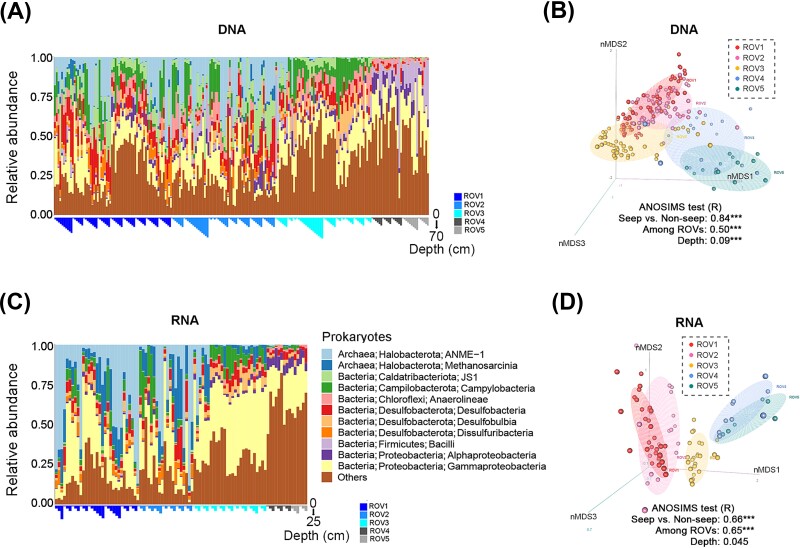
Community structure of prokaryotes. Relative abundances of sequences were shown at the class level for both DNA and RNA communities, with a shared legend of prokaryotic community compositions (A and C). Community compositional difference (at amplicon sequence variant [ASV] level) among remotely operated vehicle habitats (ROVs) were shown in the non-metric multidimensional scaling (NMDS) maps and tested for the effects from habitat type (i.e. seep and non-seep), sub-habitat (i.e. ROVs) and sample depth by ANOSIM (B and D).

For prokaryotic communities (16S), the compositional difference between seep (ROV1, 2 and 3) and non-seep (ROV4 and 5) region (ANOSIM-R = 0.86 and 0.66 in DNA and RNA communities, respectively) was much larger than that between DNA and RNA communities (ANOSIM-R = 0.19) (Fig. 3). ANME1 (*Halobacteria*, *Archaea*), *Methanosarcinia* (*Halobacteria*, *Archaea*) and *Campylobacteria* (*Campilobacterota*, *Bacteria*) had higher proportions in the seep region than the non-seep region (Fig. S4). Particularly, ANME1 was the most abundant in the active seep region, ROV1 and ROV2 (DNA: 16.70%, RNA: 28.63%, on average). In addition, *Gammaproteobacteria* sequences were abundant in most samples, with relatively higher proportions in RNA communities (DNA: 16.94%, RNA: 27.85%, on average). In contrast, *JS1* and *Bacilli* sequences had relatively higher percentages in the DNA communities (on average of 6.60% and 4.56%, respectively) than RNA communities (on average of 0.30% and 0.04%, respectively), indicating their relatively lower activities.

For both microeukaryotic and prokaryotic communities, sampling depth had consistently minor effects on the variations of community compositions, reflected by the small ANOSIM-R values (Figs 2 and 3).

### Diversity pattern and regulating factors of microbial communities

**Figure 4:**
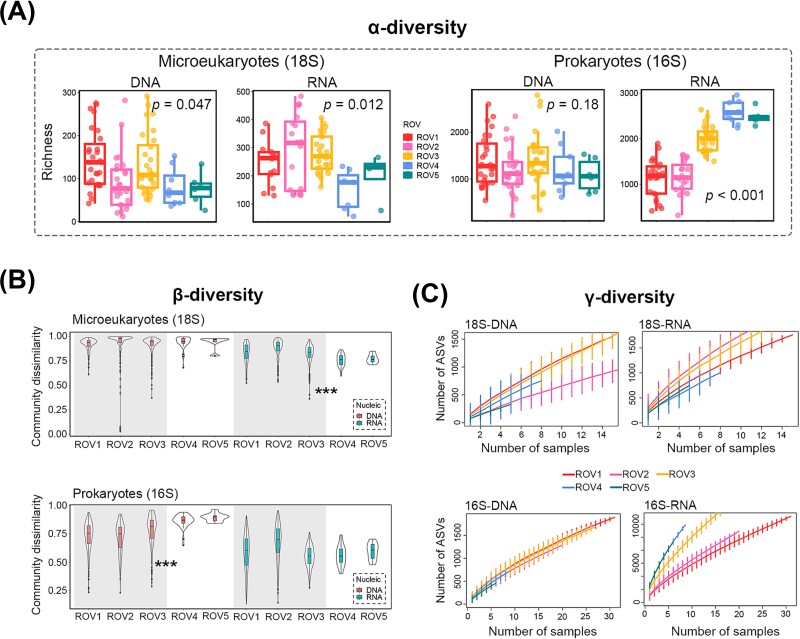
Comparison of α-, β- and γ-diversity between seep and non-seep regions in remotely operated vehicle habitats (ROVs). Wilcoxon tests were performed between seep (ROV1, 2 and 3) and non-seep (ROV4 and 5) regions, with *P* < .05 showing the significant difference (A) or using significance code: ^*^: *P* < .05, ^**^: *P* < .01, ^***^: *P* < .001, with grey-filled region indicating seep samples and white region indicating non-seep samples (B). Amplicon sequence variant (ASV) accumulation curve (with error bar) for each habitat (ROV) to compare the γ-diversity (totally detected ASV richness) with an equal number of samples (randomly selected) among habitats (C).

Higher α-diversity of microeukaryotes, represented by indices of richness, Chao1 and Faith’PD, were observed in the seep regions (ROV1, 2 and 3) than the non-seep regions (ROV4 and 5) for both DNA (except Chao1 index) and RNA communities (Wilcoxon test, *P* < .05). In contrast, a much lower α-diversity of prokaryotes was observed in the RNA communities of the seep region (especially the active seep ROV1 and 2) than the non-seep region (Wilcoxon test, *P* < .001), while there was no significantly regional difference in the DNA communities (Wilcoxon test, *P* > .05) (Fig. 4A). Regarding the compositions of richness (ASV level), the increased microeukaryotic richness in the seep region was mainly from the abundant groups, such as *Apicomplexa* in the DNA communities and *Breviatea* in the RNA communities (Fig. S5a). On the contrary, the large decrease in prokaryotic richness in the seep RNA communities was mostly from other groups which were not abundant in sequences (Fig. S5b), indicating the loss of rare or intermediate taxa, which may contribute to the significantly lower evenness of α-diversity (Fig. S6).

Spearman correlation analyses showed that environmental factors had stronger effects on the α-diversity (richness here) of RNA communities than DNA communities, for both microeukaryotes and prokaryotes (Fig. S7a). Distinct response patterns of richness to environmental factors were found between microeukaryotic and prokaryotic communities. For instance, proportions of organic carbon and total nitrogen (%OC and %TN) significantly promoted microeukaryotic richness in the RNA communities (R = 0.45 and 0.41, both *P* < .05, respectively) (Fig. S7b). Several factors, including CH_4_, DIC, -δ^13^C, -δ^15^N, DOM_BIX (biologic contribution index of DOM) and DOM_FI (fluorescence index of DOM), had strong negative correlations with prokaryotic richness in the RNA communities (Fig. S7c).

In terms of β-diversity, higher microeukaryotic diversity was found in the seep region (compared to the non-seep region) in the RNA communities while the pattern was opposite in the prokaryotic DNA communities (by Wilcoxon test, *P* < .001) (Fig. 4B). Results from phylogenetic turnover (βNTI based) showed that ecological processes shaping the microeukaryotic beta-diversity (i.e. community structure and similarity pattern, Fig. 2 and 3) were mainly stochastic processes (|βNTI| < 2, such as dispersal and drift) (Fig. S8a). Significantly higher niche breadth of microbial communities was observed in the seep region than non-seep in the microeukaryotic RNA communities (Wilcoxon test, *P* < .001) (Fig. S9). In the prokaryotic DNA communities, lower βNTI values were observed in the seep region (compared to non-seep), indicating the increasing importance of homogeneous selection and explaining the higher beta-diversity there. In the prokaryotic RNA communities, the βNTI values were mostly below −2, indicating the predominance of homogeneous selection (Fig. S8b), which reduced the community dissimilarity (Fig. 4B).

Redundancy analysis (RDA) showed that environmental factors explained more of the compositional variations in prokaryotic communities (total explanation of 49.51% and 56.74% in DNA and RNA, respectively) than that in microeukaryotic communities (total explanation of 39.69% and 42.77% in DNA and RNA, respectively) (Fig. S10). Several factors, particularly δ^13^C, DIC and DOM_BIX, had strong effects (R^2^ > 0.4, by “envfit” test) on the prokaryotic community compositions (Table S2).

At a regional scale, the seep region (compared to non-seep) had significantly higher microeukaryotic and lower prokaryotic γ-diversity, both of which were observed in the RNA communities (Fig. 4C). There was reflected in the results that ROV1, ROV2 and ROV3 had relatively higher numbers of ASV than ROV4 and ROV5 with an equal number of sites in the microeukaryotic RNA communities, while the pattern was opposite in the prokaryotic RNA communities.

In general, microeukaryotic communities had higher diversity in the seep regions than the non-seep regions while prokaryotic communities showed the opposite pattern, though the trend was not consistently significant between DNA and RNA signatures (Table S3).

### RA and seep-active groups

**Figure 5:**
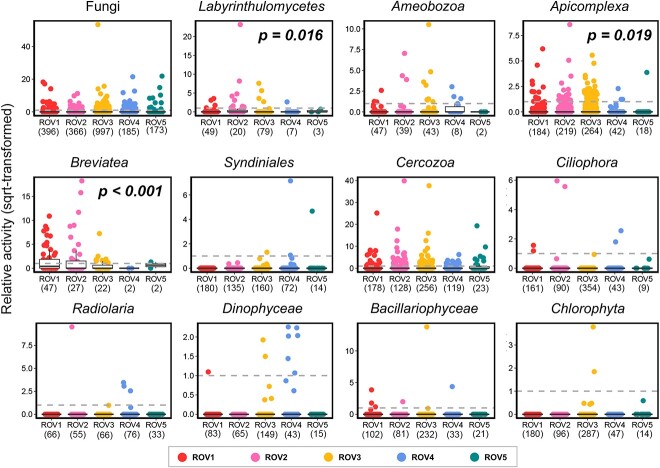
Relative activity and seep-active groups of microeukaryotes. Only abundant groups (in accord to their potential trophic modes, n = 12) are shown, with each dot representing an amplicon sequence variant (ASV). Number of total ASVs is marked below the label of ROV. A horizontal dashed line indicates the relative activity value of 1. Wilcoxon test was performed on the RA between seep (ROV1, 2 and 3) and non-seep (ROV4 and 5) regions, with *P* < .05 showing the significant difference.

For microeukaryotes, most of the ASVs (13 395 of 14 518) had a RA of zero, indicating their inactive status (Fig. 5). *Apicomplexa*, *Breviatea* and *Labyrinthulomycetes* showed significantly higher RA in the seep region than the non-seep region (Wilcoxon test, *P* < .05), regarded as seep-active groups.

**Figure 6:**
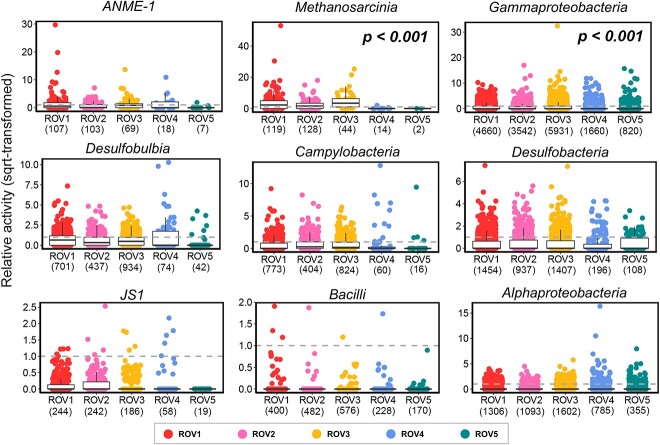
Relative activity and seep-active groups of prokaryotes. Only abundant groups are shown (n = 9), with each dot representing an amplicon sequence variant (ASV). Number of total ASVs is marked below the label of ROV. A horizontal dashed line indicates the relative activity value of 1. Wilcoxon test was performed on the RA between seep (ROV1, 2 and 3) and non-seep (ROV4 and 5) regions, with *P* < .05 showing the significant difference.

In contrast, most of the prokaryotic ASVs (101 480 of 126 503) had a RA value over zero, indicating their active status (Fig. 6). Compared to non-seep region, *Methanosarcinia* and *Gammaproteobacteria* had significantly higher RA in the seep regions (Wilcoxon test, *P* < .05), representing the seep-active groups there. ANME1 had several ASVs with quite high activity (square-rooted RA > 10) in the seep region, however, the difference (of RA) compared to non-seep region was not significant (*P* = 0.328, by Wilcoxon test). Considering the predominance of ANME1 in the prokaryotic communities (both DNA and RNA) and its much higher abundance in the seep samples than in the non-seep samples at the class level (Fig. S4), we also included it into the seep-active groups for further analyses.

### Correlations between environmental factors and the diversity of seep-active groups and whole communities

**Figure 7:**
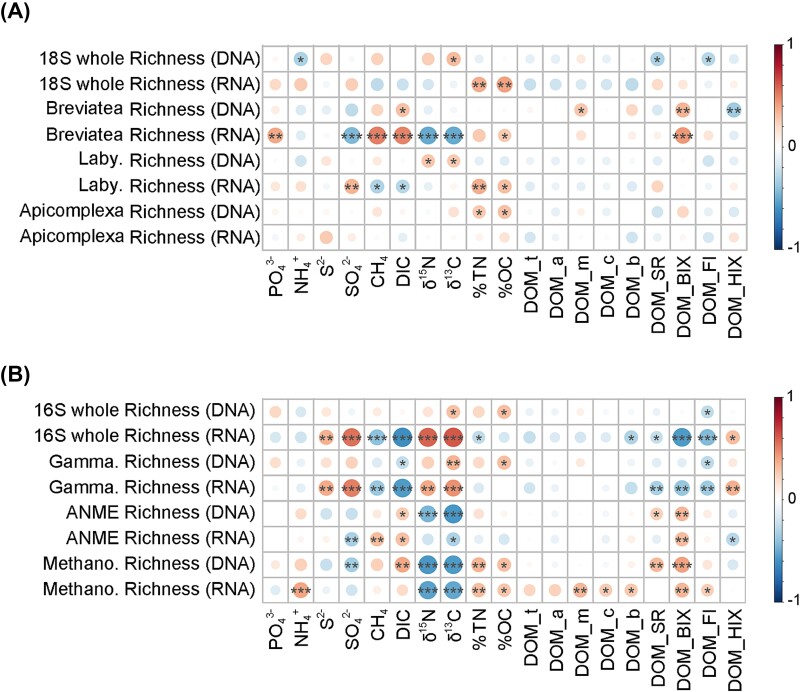
Correlations between environmental factors and the richness of seep-active groups and the whole microbial communities. Spearman correlations were used, with significance code: ^*^: *P* < .05, ^**^: *P* < .01, ^***^: *P* < .001. Laby.: *Labyrinthulomycetes*, gamma.: *Gammaproteobacteria*, Methano: *Methanosarcinia*. Abbreviations of environmental factors are in Table S1 and Metadata file 1.

In general, environmental factors had stronger influences on the α-diversity (richness here) of prokaryotic communities than that of microeukaryotic communities (Fig. 7). Seep-active microeukaryotic groups, especially *Breviatea* and *Labyrinthulomycetes*, were more influenced by environmental factors than the whole community. For instance, environmental factors, SO_4_^2−^, CH_4_, DIC, δ^13^C and δ^15^N, had strong effects (Spearman correlation R > 0.4 or < −0.4, *P* < .001) on the richness of *Breviatea* RNA communities while their effects on the whole microeukaryotic communities were relatively weaker (Fig. 7A). Notably, while *Gammaproteobacteria* and the whole prokaryotic community (especially the RNA part) showed the similar correlation patterns with environmental factors (e.g. positive to S^2−^, SO_4_^2−^, δ^15^N and δ^13^C while negative to CH_4_, DIC and DOM characters), which was opposite to the correlations between ANME1, *Methanosarcinia* and environmental factors (e.g. negative to S^2−^, SO_4_^2−^, δ^15^N and δ^13^C while positive to CH_4_, DIC and DOM character) (Fig. 7B), indicating the different responses of these prokaryotic groups to environment variations and potential cooperations or competitions among them. Besides, DOM characters, particularly DOM_BIX, had higher effects than DOM compositions (which were mostly not significant) on regulating the richness of prokaryotes (Fig. 7B).

Similar patterns were also found in the correlations between community composition (β-diversity, based on ASV relative abundance) and environmental factors, showing the stronger effects from environmental factors on prokaryotes than microeukaryotes, on seep-active groups than the whole communities and on DOM characters than compositions (Table S4).

**Figure 8:**
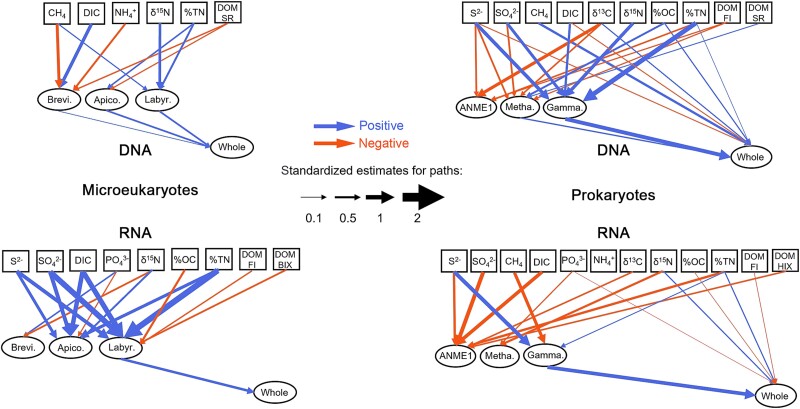
Structural equation models showing the effects of environmental factors on the richness of seep-active groups and the whole microbial communities. Only paths with significant effects (*P* < .05) were shown. Metha, *Methanosarcinia*; Gamma, *Gammaproteobacteria*; Brevi, *Breviatea*; Apico, *Apicomplexa*; Labyr, *Labyrinthulomycetes*.

Overall, all SEMs showed good fitness to the original data, with Chi-squared test *P* > .05, GFI close to 0.9 and CFI > 0.9 (Fig. 8, Table S5). The results from SEMs showed that (1) Environmental factors had greater influences on the seep-active groups than the whole community, for all the types of communities; (2) Environmental factors (such as S^2−^, CH_4_, DIC, δ^13^C, %OC and %TN in the 16S DNA communities, PO_4_^3−^, δ^15^N, %OC, %TN and DOM_FI in the 16S RNA communities) can directly influence the whole community of prokaryotes, while they indirectly influenced the whole community of microeukaryotes through seep-active groups (i.e. *Breviatea*, *Apicomplexa* and *Labyrinthulomycetes*) (Fig. 8).

## Discussion

### Interpretation of DNA and RNA communities on biodiversity and biogeochemical cycles

Our results revealed a drastic difference in community structure between DNA and RNA signatures, highlighting the importance of appropriate interpretation of them. We observed that most of the ASVs in dominant groups of microeukaryotes had extremely low relative activity (average value close to zero), suggesting their dead or dormant status and low contribution to ecosystem functioning (e.g. C, N fixation and decomposition). However, we cannot rule out their contribution to biogeochemical cycles as themselves can be important resources. This is supported by the significant correlation between the relative abundance of microeukaryotic DNA communities and organic C and N content in the sediment (Fig. 7A).

The RNA communities reflected the putatively active taxa and should be more sensitive to environmental factors, as demonstrated by our results from both richness and relative abundance aspects (Fig. 7 and Table S4). For instance, we observed that samples were more closely clustered by habitats (based on Bray–Curtis distance) and environmental factors explained more community variations (RDA analysis) in the RNA communities than the DNA counterpart for both prokaryotes and microeukaryotes. On one hand, we found that relative abundance of several heterotrophic protists, such as *Breviatea* and *Cercozoa*, were much higher in the RNA communities compared to DNA, suggesting their strong activity and potential key roles in the biogeochemical cycles [2, 66]. On the other hand, we showed that relative abundances of several groups, such as Fungi (18S) and *JS1* (16S), were dominant in the DNA communities, in accordance with previous studies [21, 23, 67]. However, their proportions were much lower in the RNA communities from our results, suggesting that most or a large part of them were inactive or dead and their roles in the ecosystem functioning might be overlooked in those studies.

One key question is whether DNA or RNA communities can better represent the true *in situ* microbial diversity. It is generally assumed that DNA detects not only living organisms but also dead cells and exogeneous DNA while RNA detects living or putatively living organisms. Therefore, lower (α-) diversity is commonly expected in RNA communities [30, 68, 69]. However, our result showed that opposite pattern, with RNA communities having higher richness than DNA communities (Figs 4 and S4) for both microeukaryotes and prokaryotes. This pattern is in accordance with several previous studies and could be explained by that higher number of RNA copies in some rare taxa (i.e. active rare taxa), making them successfully amplified and detected [70, 71], while the DNA of predominant organisms (e.g. metazoans, dinoflagellates and ciliates with high gene copy numbers) may mask the detection of rare organisms during PCR amplification [72, 73]. This could be possible in our study given the predominance of several groups (e.g. Fungi) in the DNA communities. Moreover, environmental RNA was shown to perform better than DNA on assessing benthic community diversity, with a higher richness revealed [72]. We argue that this contradictory result of richness estimation between using DNA and RNA will not influence our comparison of diversity between the seep and non-seep regions, as the pattern (i.e. higher microeukaryotic but lower prokaryotic diversity in the seep regions) was consistent between DNA and RNA communities here. Regarding the different performance and interpretation of DNA and RNA metabarcoding, we highlight the importance of combining them for estimating and comparing of microbial biodiversity, especially in the sediment, which will provide more convincing patterns and comprehensive interpretations [73].

### Cold-seep sediments promote microeukaryotic diversity but reduce prokaryotic diversity

Given the lack of comprehensive study of microbial diversity and the large spatial heterogeneity of microbial communities in the cold-seep sediment [16, 25], we compared their diversity at different scales, i.e. α (local), β (between local communities within a region) and γ-diversity (regional) between seep regions and non-seep regions. Our results showed that, in general, microeukaryotic diversity was promoted in the cold-seep sediment while prokaryotic diversity was reduced.

Here we showed that prokaryotic communities were more influenced by environmental selection compared to microeukaryotic communities in the cold-seep sediment. This was reflected by the more contribution of homogeneous selection (i.e. βNTI < −2), together with the higher total explanation of environmental factors to the variations in prokaryotic communities. We attribute the dominance contribution of homogeneous selection in the seep regions to the high concentration of several environmental factors, such as CH_4_, S^2−^, DIC, and organic matter. This is supported by the findings from previous studies showing that β-diversity of bacterial communities was significantly affected by concentrations of SO_4_^3−^, H_2_S, and DIC in the cold-seep sediment of Eastern Mediterranean Sea [16] and South China Sea [74].

In contrast, microeukaryotic communities were mainly shaped by stochastic processes (dispersal and ecological drift), with minor effects from selection processes. This pattern was also observed in the sediment of cold seeps [25], estuary [75] and river [76], suggesting that sedimental microeukaryotes, compared to that in the water, are less sensitive to environmental factors. We attribute their low influence from environmental selection in the DNA communities to the inactive or dead status of the most microeukaryotic ASVs as reflected by their relative activity, which will decouple or mismatch the correlation between taxa relative abundance and phylogenetic distances used for calculating phylogenetic turnover and ecological processes. For their RNA communities, we found significant higher niche breadth of microeukaryotes at the seep region compared to the non-seep region (Fig. S9). Organisms with wider niche breadth tend to be less affected by environmental selection, explaining the high contributions of stochastic processes [62]. Here, the wider niche breadth of microeukaryotes at seep region can be contributed by the different trophic modes of these active groups, such as parasitism in *Apicomplexa*, heterotrophs in *Breviatea*, and saprotrophs in Fungi and *Labyrinthulomycetes*, which could increase the resource (e.g. organic matter) use efficiency, reduce competition, and promote species coexistence.

### Seep-active groups are more important than the whole community in the cold-seep sediment

RA, calculated as the ratio of the relative proportion of sequences in the DNA community to that in the RNA community) can vary greatly among different microbial groups due to their unique traits [37], and the ratios can be influenced by environmental factors, such as water column depth [38, 77]. In this study we identified several key microbial groups that were more active in the seep regions than non-seep regions, based on their RA. We showed that these groups were not only important in terms of their high abundance and activity, but also contributed greatly to the biodiversity (at community level) and biogeochemical cycles in the cold-seep sediment.

In the microeukaryotic communities, *Breviatea* (Obazoa), *Apicomplexa* (Alveolata), and *Labyrinthulomycetes* (Opisthokonta), with different trophic modes, were representative active groups in the cold-seep sediments. *Breviatea* is a group of basal eukaryotes that contains putatively anaerobic organisms, which has only been recovered from environmental DNA sequencing [2, 6, 78, 79]. Relatively higher abundance of *Breviatea* sequences were detected in the active seep sediments than inactive sediments, particularly in microbial mats, suggesting their key roles in anerobic habitats [2]. Together with other groups, such as *Ciliophora* and *Cercozoa*, which were abundant in the RNA communities, they could be important bacterial grazers, contributing greatly to the microbial food webs in the seep sediment [80]. *Labyrinthulomycetes* [81] are important decomposers of organic matter in the marine sediment [82], playing key roles in the detrital decomposition in the marine upper sediment layer [19]. Our results highlighted their similarly important roles in the seep sediments, which could also be reflected by the high correlations between their relative abundance and environmental factors, such as NH_4_^+^ (which could be release from decomposition of detritus), organic carbon (%C) and nitrogen (%N), and DOM composition and characters (Table S4). In particular, their communities had strong correlations with δ^13^C and δ^15^N, which were even higher than the whole community (DNA), suggesting their key roles in the contribution to biogenic organic matters. *Apicomplexa* are a large phylum of mainly parasitic alveolates, which were detected or reported as abundant groups in previous studies of seep sediments [2, 24, 83, 84]. In our study, their relative proportions of sequences were higher in DNA communities than RNA communities, a similar pattern as report by Massana *et al*. [37], which could be due to their high genomic rDNA copy number as parasites.

In the prokaryotic communities, archaea ANME-1 and *Methanosarcinia* (mainly ANME-2 and ANME-3) have been widely reported to conduct the anaerobic oxidation of methane in the cold-seep sediment, contributing greatly to the biological sink of methane [74, 85–87]. *Gammaproteobacteria*, containing many methanotrophs and sulphur-reducing bacteria, was the most abundant group in the global methane seeps [3, 87], and responsible for the community differences between cold seep and other habitats [9]. It also contributed the most to the total richness of prokaryotic communities in our study, as reflected by SEMs. Similar to our results, a recent study showed that methane-metabolizing archaea and sulphate-reducing *Gammaproteobacteria* were more abundant in the RNA communities than DNA communities, displaying niche differentiations among their subgroups [88]. Interestingly, from the SEMs, we found that while most environmental factors negatively affected the richness of ANME1 and *Methanosarcinia*, *Gammaproteobacteria* could increase their richness under the high concentrations of these factors. This could be due to the high diversity of *Gammaproteobacteria* and their different responses to environmental factors, which suggest that further analyses on their sub-groups should be conducted.

Our results suggest that several seep-active microbial groups may play more important roles than the entire community on the biodiversity and biogeochemical cycles in the cold-seep sediments. This finding was supported by the higher correlations between the seep-active groups and environmental factors compared to the whole communities (e.g. relative abundance of *Gammaproteobacteria* in both DNA and RNA communities; *Breviatea* richness in RNA communities) (Fig. 7, Table S4). We further showed that the importance of seep-active groups was much greater in the microeukaryotic communities than the prokaryotic communities. This was reflected by the SEMs where environmental factors can directly affect the whole prokaryotic richness, while they can only indirectly regulate the whole microeukaryotic richness with a first step on the seep-active groups. Together with the more prevalent paths in prokaryotic communities, the results from SEMs supported our finding that prokaryotic communities were more influenced by environmental selection than microeukaryotic communities in the cold-seep sediments.

It should be noted that the definition and comparisons of seep-active groups in our study were mainly conducted at the group level (i.e. class or phylum levels), while different insights might be obtained with analyses performed at finer levels or using different comparison methods. Thus, we cannot rule out the potential key roles of other groups which were not defined as seep-active groups in our study. We also acknowledge the limitations of our findings (e.g. diversity trends), which may come from the lack of global-scale comparison (i.e. other seep regions), and insufficient number of control non-seep regions.

In conclusion, we comprehensively investigated the microbial diversity at α, β and γ scales in the Haima cold seep and found that microeukaryotes showed an increasing trend in diversity, while prokaryotes displayed a decreasing trend. These findings could be explained by the discrepancy in the effects of environmental factors on microbial richness and key ecological processes regulating the community structures between microeukaryotes and prokaryotes. These results will deepen our understanding of the biodiversity from different scales, which is crucial for carrying out spatial management of biodiversity conservation. Moreover, we observed that although DNA and RNA communities displayed similar diversity trends, they differed significantly in composition, correlations with environmental factors, and contributions to biogeochemical cycles. This highlights the importance of combining both DNA and RNA approaches when studying microorganisms in the cold seep. We further identified several seep-active groups within the microbial communities and showed they were more sensitive to environmental variables and played central roles in regulating whole- community diversity and ecosystem functions. Together with the finding that most of the ASVs in the microeukaryotic communities had an RA of zero, suggesting their dead or inactive status, our results, from the activity aspect, support the notion that “only few microbial taxa at seeps potentially impact the global carbon budget today” [3].

## Supplementary Material

Supplementary_figures_and_tables_ycaf002

Metadata_file1_ycaf002

## Data Availability

Raw sequencing reads for analyses in this study were deposited in online open database of National Center for Biotechnology Information Search database (NCBI) with projection accession number of PRJNA1097764. R scripts used for analysis can be found from the website: https://github.com/xzhimenghkust/Haimap-cold-seep-microbial-diversity
