# Supplementary material for: Contrasting diversity patterns between microeukaryotic and prokaryotic communities in cold-seep sediments
Source: ISME Commun. 2025 Jan 8;5(1):ycaf002. doi: 10.1093/ismeco/ycaf002 (PMC11879339; doi:10.1093/ismeco/ycaf002)
Supplement: Supplementary_figures_and_tables_ycaf002 [file supplementary_figures_and_tables_ycaf002.pdf]

Contrasting diversity patterns between microeukaryotic and prokaryotic communities in the cold-seep sediments

Zhimeng Xu[1], Jiawei Chen[1], Wenzhao Liang[1], Zhao Liang Chen[1], Wenxue Wu[2], Xiaomin Xia[3,4], Bingzhang Chen[5], Ding He[1], Hongbin Liu[1,*]

[1]Department of Ocean Science, The Hong Kong University of Science and Technology, Hong Kong, China.

[2]State Key Laboratory of Marine Resource Utilization in South China Sea, Hainan University, Haikou, China

[3]Carbon Neutral Innovation Research Center and State Key Laboratory of Marine Environmental Science, Xiamen University, Xiamen, Fujian, 361102, China

[4]Key Laboratory of Tropical Marine Bio-resources and Ecology, South China Sea Institute of Oceanology, Chinese Academy of Sciences, Guangzhou, China

[5]Department of Mathematics and Statistics, University of Strathclyde, Glasgow, UK

Corresponding to: Hongbin Liu

Email: liuhb@ust.hk

Address: Department of Ocean Science, The Hong Kong University of Science and Technology, Hong Kong, China.

Running title: microbial diversity in cold seeps

Supplementary files

Supplementary figure: n = 10
Supplementary tables: n = 5

Supplementary figures

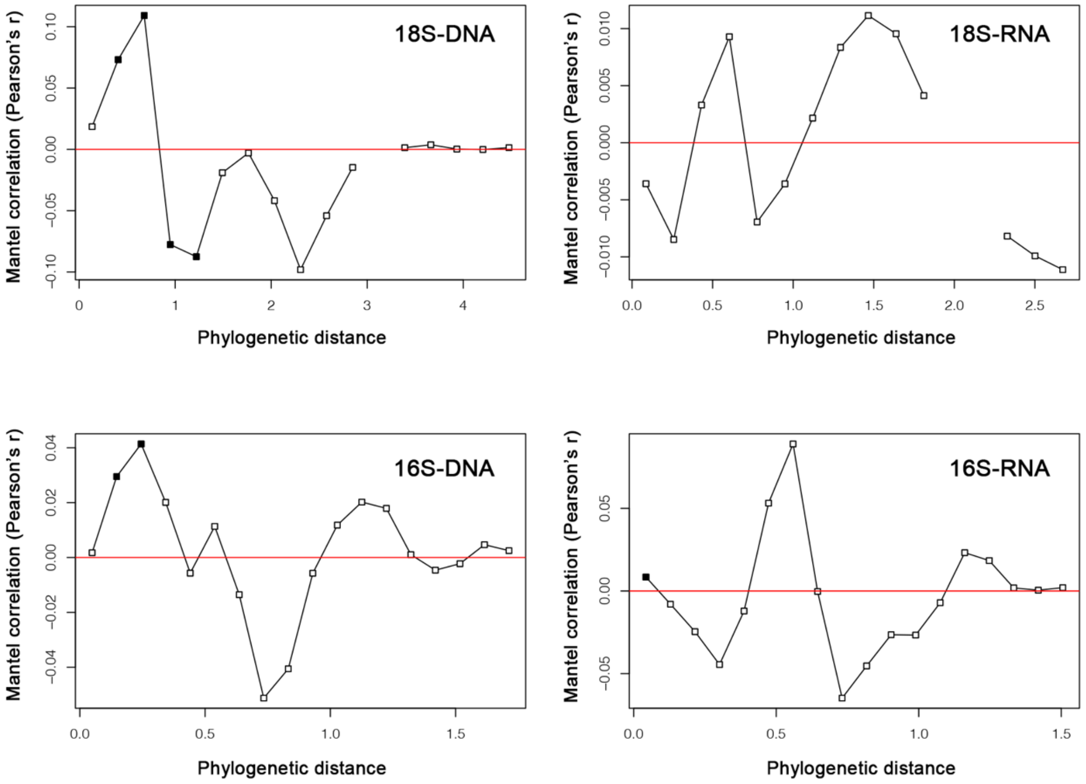

Figure S1 | Testing of phylogenetic signal across short phylogeny distance. A Mantel correlogram was used to test the correlation between ASV niche difference and ASV phylogenetic difference. Optimal environmental factors (all factors together) for ASVs in each phytoplankton community assembly were calculated as their niches. he test was conducted by the "mantel.correlog" function in the vegan package. Filled and open symbols represent significant and nonsignificant correlations, respectively.

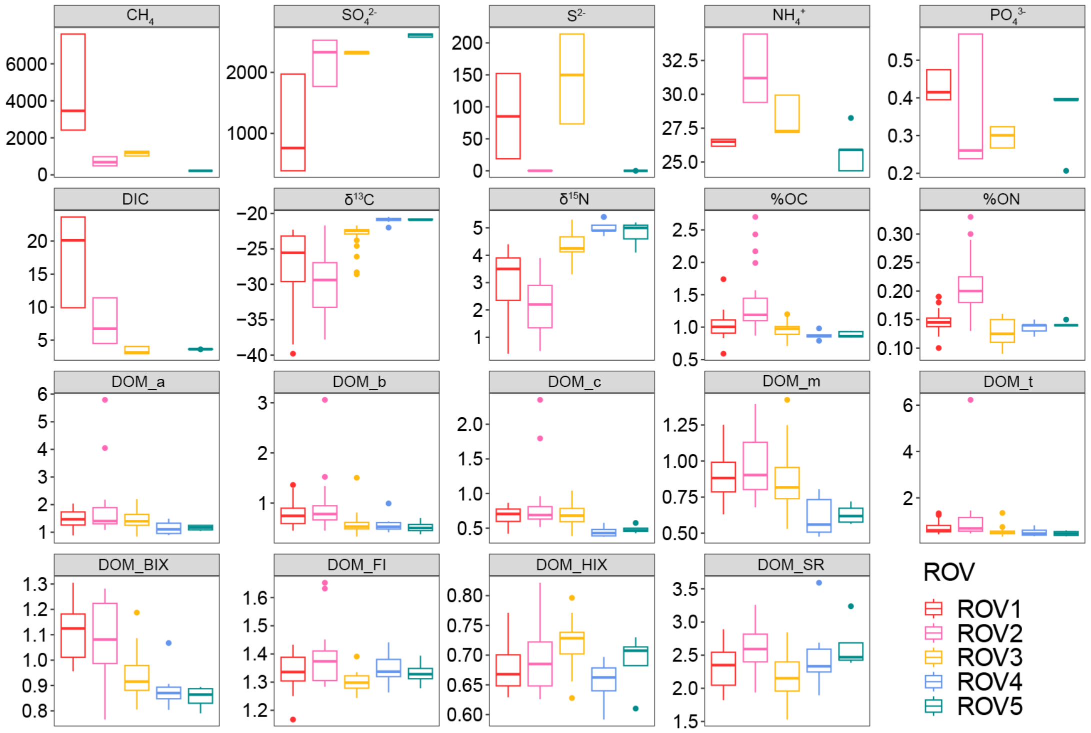

Figure S2 | Distributions of environmental factors. Parameters were measured with sediment samples from 0 - 10 centimeter below surface (cmbs). Detail values, units and abbreviations are in Table S1 and Metadata file 1.

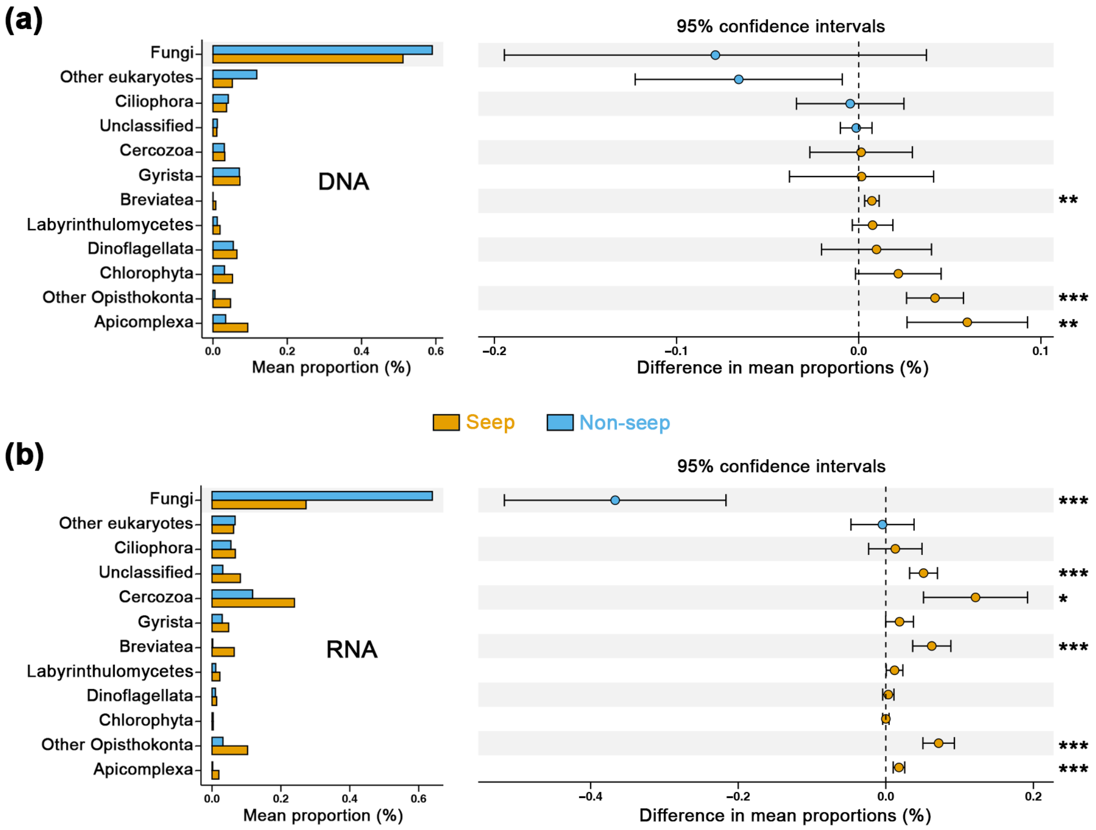

Figure S3 | Differential distribution of microeukaryotic groups between seep and non-seep regions. Mean relative abundance of each microeukaryotic group was shown at the class level and statistically compared between seep (orange) and non-seep (blue) samples for both DNA **(a)** and RNA **(b)** communities. Corrected *p*-values were calculated using "Bonferroni" approach, with significance codes: *: $p < 0.05$; **: $p < 0.01$; ***: $p < 0.001$.

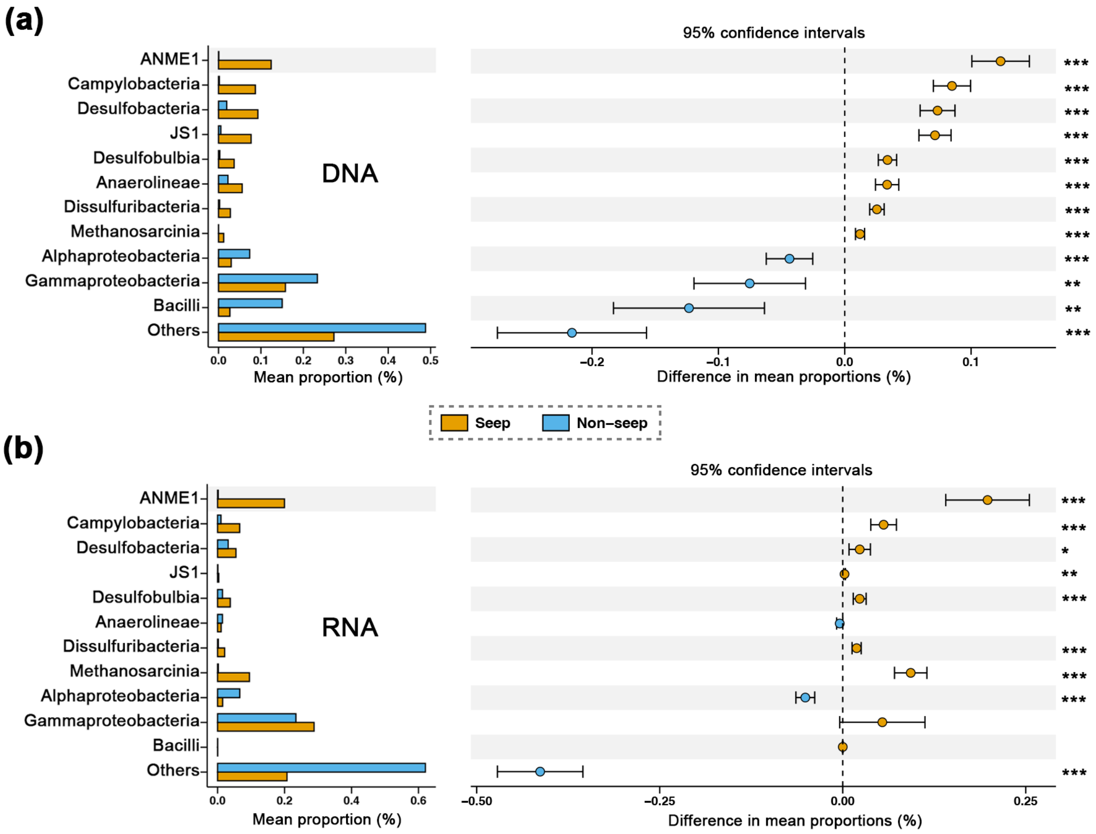

Figure S4 | Differential distribution of prokaryotic groups between seep and non-seep regions. Mean relative abundance of each prokaryotic group was shown at the class level and statistically compared between seep (orange) and non-seep (blue) samples for both DNA **(a)** and RNA **(b)** communities. Corrected *p*-values were calculated using “Bonferroni” approach, with significance codes: *: $p < 0.05$; **: $p < 0.01$; ***: $p < 0.001$.

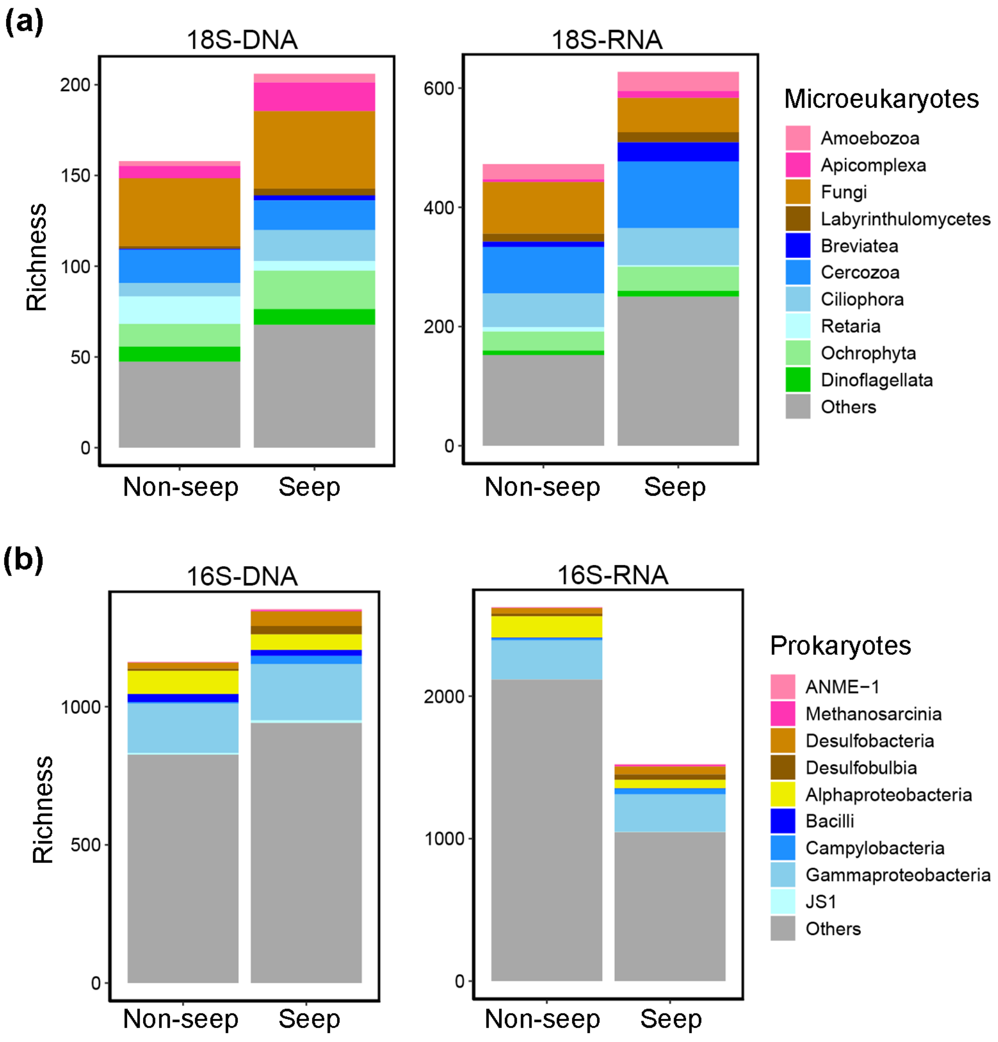

Figure S5 | Richness of abundant groups in microbial communities. ASV richness of each abundant group (relatively abundant in sequence number) was shown for both microeukaryotic (18S DNA and RNA) **(a)** and prokaryotic communities (16S DNA and RNA) **(b)**, with comparisons between non-seep and seep samples.

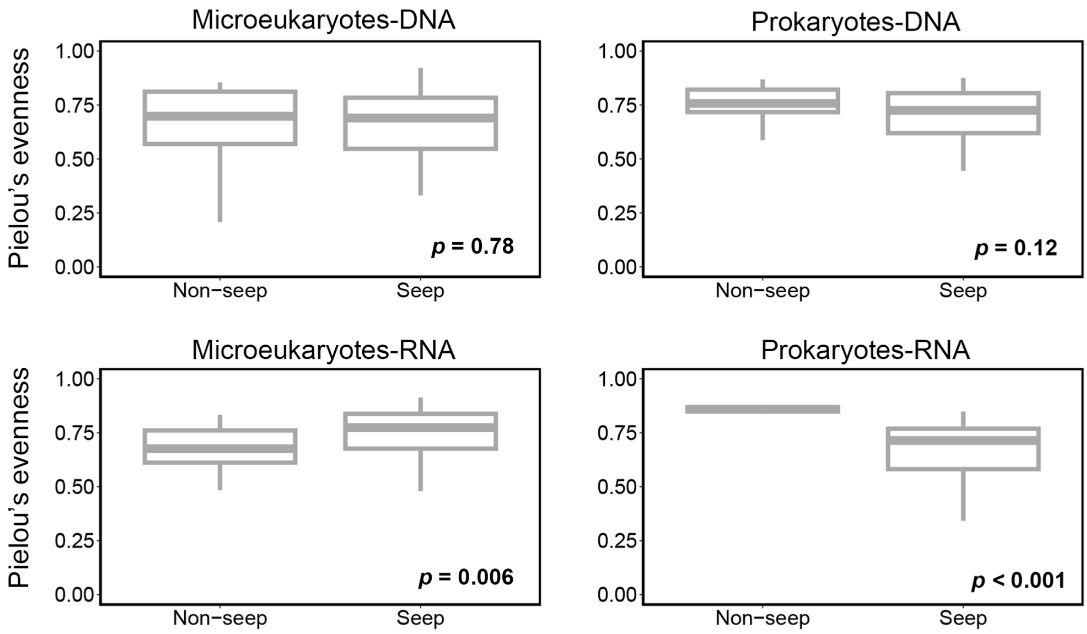

Figure S6 | Evenness of microbial communities. Pielou's evenness was calculated at the ASV level for microeukaryotic and prokaryotic communities. Comparison was made between samples from non-seep and seep regions with significant difference tested by Wilcoxon test.

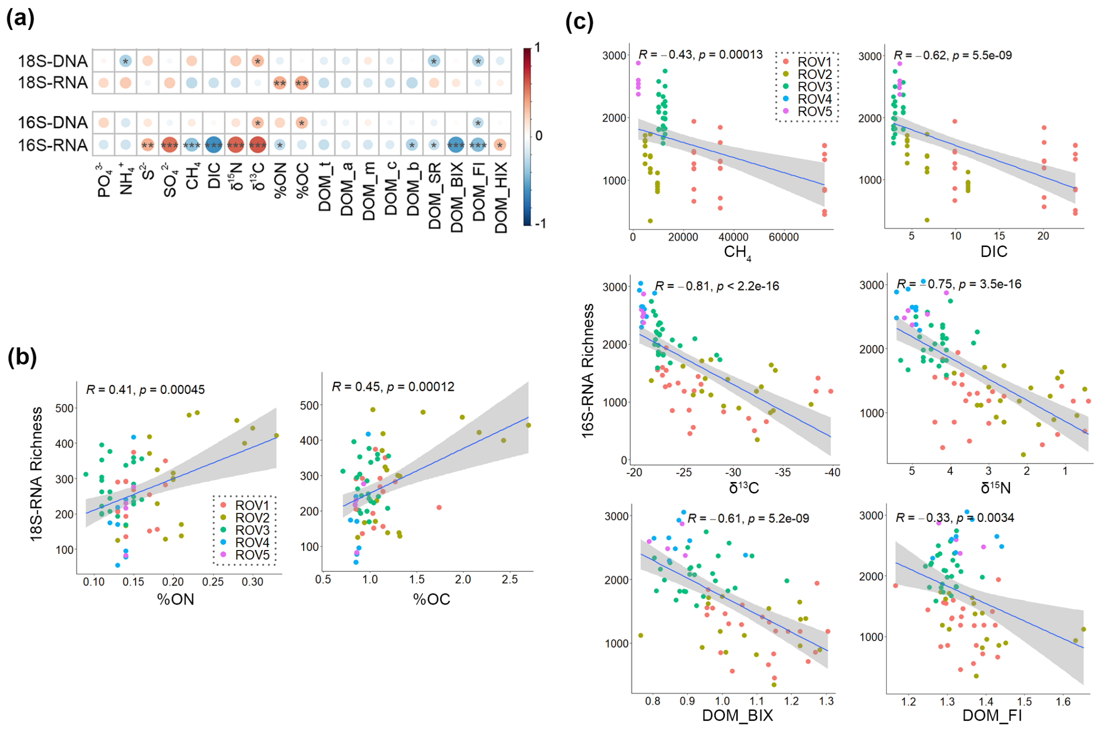

Figure S7 | Effects of environmental factors on the microbial richness. (a) shows the Spearman's rank correlation coefficient (R) between environmental factors and microbial community richness. R close to 1 (red) means strong positive correlation while R close to -1(blue) means strong negative effects**.** Distinct correlation patterns were shown for the effects from environmental factors on the richness between microeukaryotic **(b)** and prokaryotic **(c)** RNA communities. Different color of dots indicates different ROV. Note that $\delta^{13}C$ and $\delta^{15}N$ are negative values.

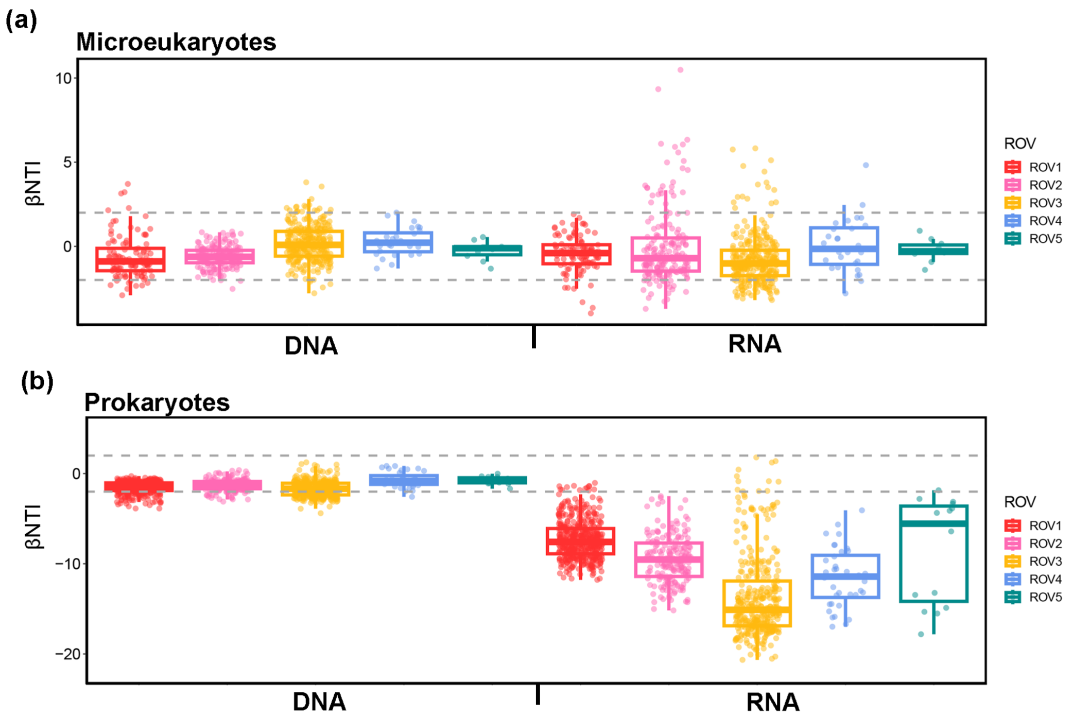

Figure S8 | Phylogenetic turnover (βNTI) in microbial communities calculated by null model. Different ecological processes were indicated by values of βNTI with βNTI > 2 indicating heterogeneous selection, βNTI < - 2 indicating heterogeneous selection and 2 < βNTI < - 2 indicating stochastic processes such as dispersal and ecological drift. βNTI = 2 or -2 are shown by two dash lines. Each dot represents a βNTI value which was calculated from any two samples from one ROV, with different colors of dots represent different ROVs.

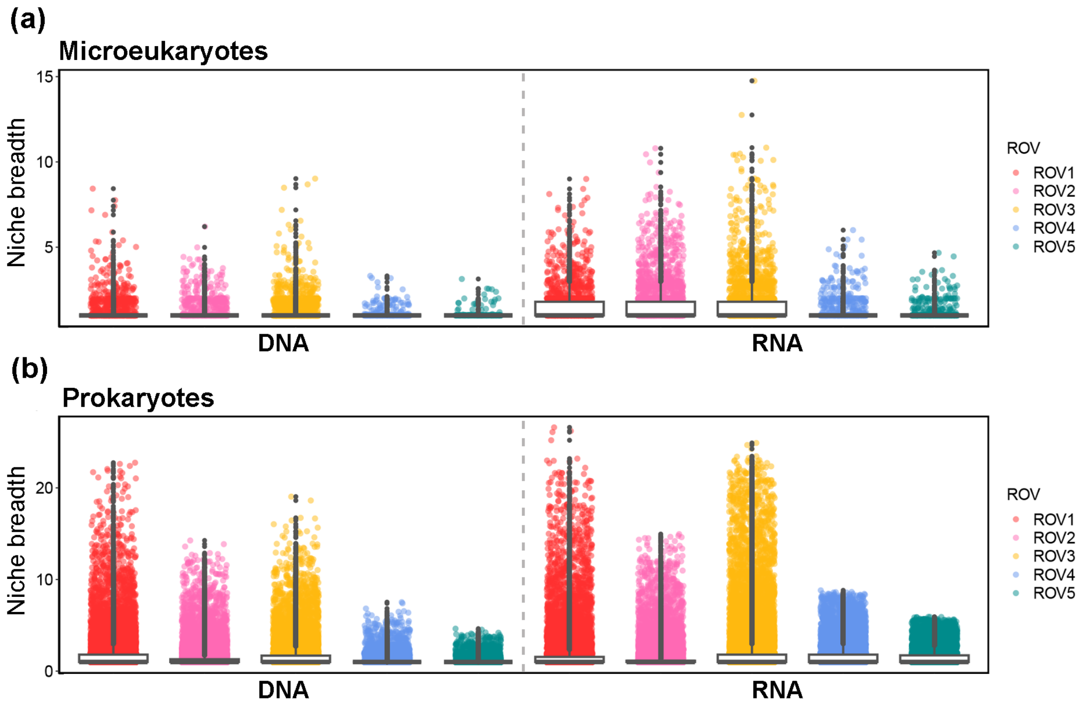

Figure S9 | Niche breadth of microbial communities in each habitat. Niche breadth of ASVs within each ROV was calculated using the Levins' Bcom index and compared between DNA and RNA signatures, as well as between seep (ROV1, 2 & 3) and non-seep regions (ROV4 & 5), for both microeukaryotes **(a)** and prokaryotes **(b)**.

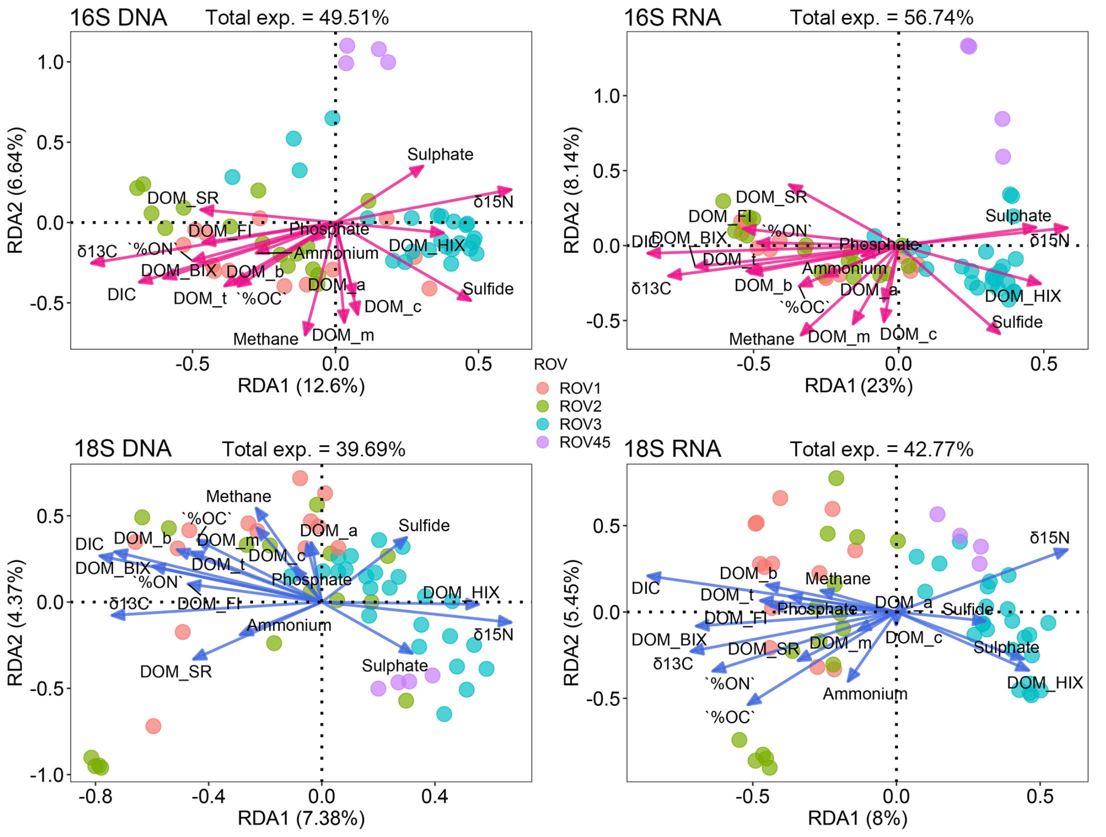

Figure S10 | Redundancy analysis (RDA) on the relationships between environmental factors and microbial community compositions. Each dot represents a microbial community, with color indicating affiliated ROV. Total exp.: total explanations from all environmental factors on community variations (ASV level). Community closer to the environmental factor (pointed by arrows) indicates a stronger positive correlation, while opposite to the arrow direction indicating a negative effect.

Supplementary tables

Table S1. Descriptions of the composition and characters of DOM.

	Ex/Em wavelength	Description
**Composition**		
**a**	Ex260, Em(380:460)	Terrestrial humic-like, high molecular weight, aromatic humic, hydrophobic acid fraction; always high in wetlands and forested environments
**b**	Ex275, Em310	Tyrosine-like organic compounds, associated with amino acids and hydrophobic neutral fraction; more degraded peptide material
**c**	Ex350, Em(420:480)	High molecular weight, humic-like compounds; high in terrestrial environments
**m**	Ex312, Em(380:420)	Low molecular weight, marine humic-like compounds
**t**	Ex275, Em340	Tryptophan-like organic compounds, associated with amino acids, hydrophobic base fraction, and hydrophilic acid fraction; less degraded peptide material
**Character**		
**BIX**		**Biological index**, which represents the relative contribution of authigenic/biogenic substances to DOM
**HIX**		**Humification index**,which positively indicates the degree of humification in the DOM
**SR**		**Spectral Slope ratio**, negatively correlated with the molecular weight of DOM
**FI**		**Fluorescence index**. The larger the value, the more recently DOM was produced.

Ex/Em: excitation/emission. More details see Wasswa et al., 2019.

Reference:

Wasswa, J., Mladenov, N., & Pearce, W. (2019). Assessing the potential of fluorescence spectroscopy to monitor contaminants in source waters and water reuse systems. Environmental Science: *Water Research & Technology*, 5(2), 370-382.

Table S2. Relative contributions of environmental factors on microbial community compositions based on redundancy analysis (RDA)

	16S-DNA		16S-RNA		18S-DNA		18S-RNA	
	R^2	*p*	R^2	*p*	R^2	*p*	R^2	*p*
**Phosphate**	0.028	0.481	0.030	0.459	0.046	0.278	0.167	0.004**
**Ammonium**	0.102	0.071.	0.091	0.076.	0.095	0.073.	0.152	0.011*
**Sulfide**	0.340	0.001***	0.379	0.001***	0.189	0.003**	0.262	0.001***
**Sulphate**	0.191	0.007**	0.231	0.003**	0.176	0.008**	0.314	0.001***
**Methane**	0.412	0.001***	0.358	0.001***	0.250	0.001***	0.207	0.005**
**DIC**	0.528	0.001***	0.600	0.001***	0.484	0.001***	0.681	0.001***
**$\delta^{15}N$**	0.454	0.001***	0.475	0.001***	0.556	0.001***	0.569	0.001***
**$\delta^{13}C$**	0.668	0.001***	0.564	0.001***	0.493	0.001***	0.474	0.001***
**%TN**	0.280	0.002**	0.196	0.012*	0.295	0.002**	0.569	0.001***
**%OC**	0.231	0.003**	0.148	0.029*	0.198	0.005**	0.504	0.001***
**DOM_t**	0.205	0.006**	0.131	0.035*	0.120	0.034*	0.127	0.030*
**DOM_a**	0.116	0.025*	0.071	0.133	0.074	0.128	0.022	0.583
**DOM_m**	0.245	0.001***	0.197	0.001***	0.203	0.003**	0.032	0.428
**DOM_c**	0.170	0.006**	0.106	0.057.	0.066	0.162	0.015	0.68
**DOM_b**	0.204	0.005**	0.175	0.012*	0.210	0.004**	0.200	0.003**
**DOM_SR**	0.205	0.003**	0.285	0.001***	0.274	0.001***	0.261	0.002**
**DOM_BIX**	0.449	0.001***	0.446	0.001***	0.625	0.001***	0.458	0.001***
**DOM_FI**	0.194	0.004**	0.244	0.006**	0.180	0.004**	0.164	0.007**
**DOM_HIX**	0.131	0.023*	0.250	0.001***	0.299	0.001***	0.252	0.001***

Relative contributions of environmental factors to variations in microbial community compositions (based on ASV relative abundance) were calculated by the “envfit” function in the R package, with the larger R^2 value indicating the greater influence. Significance codes: ***: $p < 0.001$; **: $p < 0.01$; *: $p < 0.05$.

Table S3. Comparison of diversity between seep and non-seep regions.

Diversity	Microeukaryotes		Prokaryotes	
	DNA	RNA	DNA	RNA
**α**	**Higher**	**Higher**	n.s.	**Lower**
**β**	n.s.	**Higher**	**Lower**	n.s.
**γ**	n.s.	**Higher**	n.s.	**Lower**

"Higher" (or "lower") means seep regions had significantly higher (or lower) microbial diversity than non-seep regions. "n.s." represents insignificant differences.

Table S4. Correlation between microbial community compositions and environmental factors

Community	DNA RNA	PO_4^{3-}	NH_4^+	S^{2-}	SO_4^{2-}	CH_4	DIC	%OC	%TN	$\delta^{13}C$	$\delta^{15}N$	DOM comp.	DOM char.
**18S**													
**Brevi.**	D	0.06	**0.10**	0.01	0.09	-0.06	-0.04	0.04	**0.09**	**0.09**	**0.13**	0.02	0.02
	R	0.04	0.06	**0.13**	0.09	0.12	0.11	-0.07	0.01	**0.18**	**0.16**	0.03	**0.11**
**Labyr.**	D	0.05	**0.19**	0.02	-0.06	-0.06	-0.07	**0.13**	**0.15**	**0.23**	**0.26**	0.10	**0.15**
	R	0.04	**0.15**	0.01	0.07	-0.02	0.06	-0.07	-0.08	**0.13**	**0.12**	0.14	**0.16**
**Apico.**	D	0.01	0.02	**0.06**	0.04	0.05	0.03	0.07	0.08	**0.17**	**0.16**	-0.02	0.04
	R	0.08	0.06	0.05	**0.14**	**0.17**	**0.14**	-0.02	-0.05	0.09	**0.09**	0.04	0.03
**Whole**	D	**0.08**	**0.13**	**0.05**	0.05	0.03	**0.06**	**0.09**	**0.15**	**0.18**	**0.20**	**0.10**	**0.22**
	R	**0.12**	**0.14**	**0.09**	0.06	0.03	**0.08**	**0.07**	**0.11**	**0.13**	**0.17**	**0.13**	**0.21**
**16S**													
**ANME-1**	D	-0.04	-0.03	**0.07**	0.02	-0.02	**0.06**	-0.03	0.01	**0.13**	**0.07**	-0.05	0.03
	R	-0.02	-0.05	**0.09**	0.05	-0.02	**0.13**	-0.07	-0.04	0.07	0.04	-0.05	**0.09**
**Metha.**	D	-0.01	-0.05	**0.09**	-0.03	-0.05	0.02	-0.07	-0.01	**0.16**	**0.16**	-0.08	0.02
	R	0.06	**0.19**	**0.09**	-0.05	-0.08	-0.02	0.01	0.06	0.07	**0.11**	0.06	0.09
**Gamma.**	D	0.07	**0.19**	**0.15**	-0.02	-0.08	0.00	0.03	**0.13**	**0.30**	**0.29**	0.03	**0.25**
	R	**0.24**	**0.24**	**0.10**	**0.21**	**0.12**	**0.26**	0.11	**0.20**	**0.39**	**0.39**	0.06	**0.21**
**Whole**	D	0.03	**0.12**	**0.13**	-0.03	-0.08	-0.01	0.01	0.09	**0.27**	**0.23**	-0.02	**0.17**
	R	**0.18**	**0.20**	**0.12**	**0.18**	0.08	**0.22**	0.08	**0.14**	**0.31**	**0.31**	**0.11**	**0.32**

Mantel tests were performed to show the effects from environmental factors on the compositions of seep-active groups and the whole communities, from both microeukaryotes (18S) and prokaryotes (16S), DNA (D) and RNA (R) aspects. Values of Mantel test R were shown in the table with heavier red color indicating stronger correlations. Significant correlations ($p < 0.05$) were shown in bold. Seep-active groups include: Brevi.: Breviatea; Labyr.: Labyrinthulomycetes; Apico.: Apicomplexa; Metha.: Methanosarcinia; Gamma.: Gammaproteobacteria. Whole: whole community. DOM comp.: DOM compositions. DOM char.: DOM characters.

Table S5. Fitness of structural equation models (SEM)

Community	Chi-sq	*p*-value	DF	GFI	RMSEA	SRMR	CFI
**16S-DNA**	31.514	0.086	22.000	0.924	0.082	0.041	0.967
**16S-RNA**	44.809	0.082	33.000	0.871	0.075	0.043	0.952
**18S-DNA**	25.330	0.088	17.000	0.850	0.088	0.077	0.905
**18S-RNA**	21.327	0.501	22.000	0.889	0.000	0.052	1.000

Chi-sq: Chi-squared of model which assesses overall fit and discrepancy between sample and fitted covariance matrices, with an acceptable *p*-value over 0.05. DF: Degree of Freedom; GFI: Goodness of Fit Index; RMSEA: Root Mean Square Error of Approximation; SRMR: Standardized Root Mean Square Residual; CFI: Comparative Fit Index.
